# Magnetic coupling mechanisms in particle/thin film composite systems

**DOI:** 10.3762/bjnano.1.12

**Published:** 2010-12-01

**Authors:** Giovanni A Badini Confalonieri, Philipp Szary, Durgamadhab Mishra, Maria J Benitez, Mathias Feyen, An Hui Lu, Leonardo Agudo, Gunther Eggeler, Oleg Petracic, Hartmut Zabel

**Affiliations:** 1 Institut für Experimentalphysik/Festkörperphysik, Ruhr-Universität Bochum, D-44780 Bochum, Germany; 2 Max-Planck Institut für Kohlenforschung, D-45470 Mülheim an der Ruhr, Germany; 3 Institut für Werkstoffe, Ruhr-Universität Bochum, D-44780 Bochum, Germany

**Keywords:** exchange bias, iron oxide nanoparticles, nanoparticle self-assembly, nanoparticle-thinfilm composite, super-spin glass interaction

## Abstract

Magnetic γ-Fe_2_O_3_ nanoparticles with a mean diameter of 20 nm and size distribution of 7% were chemically synthesized and spin-coated on top of a Si-substrate. As a result, the particles self-assembled into a monolayer with hexagonal close-packed order. Subsequently, the nanoparticle array was coated with a Co layer of 20 nm thickness. The magnetic properties of this composite nanoparticle/thin film system were investigated by magnetometry and related to high-resolution transmission electron microscopy studies. Herein three systems were compared: i.e. a reference sample with only the particle monolayer, a composite system where the particle array was ion-milled prior to the deposition of a thin Co film on top, and a similar composite system but without ion-milling. The nanoparticle array showed a collective super-spin behavior due to dipolar interparticle coupling. In the composite system, we observed a decoupling into two nanoparticle subsystems. In the ion-milled system, the nanoparticle layer served as a magnetic flux guide as observed by magnetic force microscopy. Moreover, an exchange bias effect was found, which is likely to be due to oxygen exchange between the iron oxide and the Co layer, and thus forming of an antiferromagnetic CoO layer at the γ-Fe_2_O_3_/Co interface.

## Introduction

Recently, the study of composite magnetic nanostructures has received great interest due to the potential applications as permanent magnets or advanced data storage media [1–5]. In particular, systems where nanoparticles (NPs) represent at least one of the constituent materials [3] have generated much attention. A large number of investigations can be found that address potential technological applications, preparation methods and fundamental properties of magnetic NPs, such as in photonics [6–7], nanomedicine [8–10], electronics [11–12] and data storage technology [13–15]. In the latter case, composites of magnetic NPs grown onto or embedded in a host matrix have received particular attention due to their potential use for hard disk drive media [13,15–17].

In most cases, magnetic NP/thin film composites are prepared by physical growth methods, such as sputtering [18–19], sequential pulsed laser deposition [20–21], sputtering gas aggregation [22] or mechanical milling [23]. In this work, we report a different approach to fabricate composite nanoparticle/thin-film materials, i.e., which combines the use of both chemical and physical growth methods. The composite material can be successfully prepared over areas larger than 100 mm^2^ and is obtained by combining chemical synthesis of the NPs, their mechanical self-assembly on top of a substrate, and ion-beam sputtering of a magnetic layer. All experimental details about the fabrication of the present system are described in the last section.

While the physical properties of magnetic NPs are well documented [24–27], the collective behavior of self-assembled magnetic NPs on the one hand and their interaction with a magnetic substrate on the other hand is less well studied. The aim of our present investigation is to shed light on these various interactions.

## Results and Discussion

### Structural characterization

**Figure 1:**
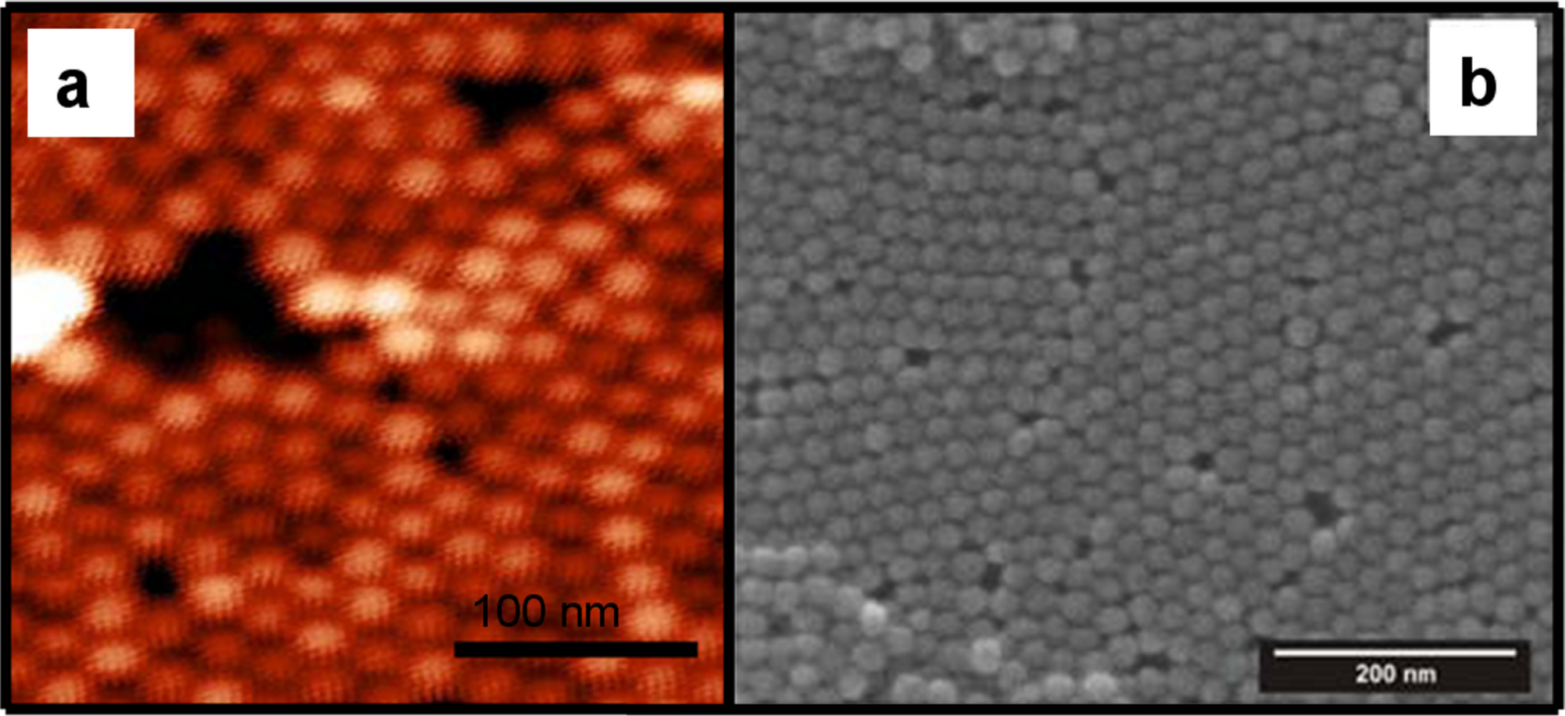
AFM (a) and SEM (b) images showing the self-assembly of the NPs in a close-packed hexagonal structure as a consequence of the spin-coating process.

Hexagonal close packing of self-assembled NPs as a result of the spin-coating process was confirmed by means of atomic force microscopy (AFM) images in Figure 1a and scanning electron microscopy (SEM) images in Figure 1b. The monodisperse nature of the particles and their ordering can be observed in these images. Furthermore, in both images common faults can be seen which are encountered in NP monolayer samples prepared by spin-coating, such as the presence of regions with two overlapping layers, missing particles (voids), and dislocations separating domains of hexagonal order. Aside from the presence of local defects, the spin-coating technique has proven to be able to produce long range hexagonal order over areas of 10 × 10 mm^2^ with a structural coherence length, as probed by scattering techniques, in the order of 200–300 nm [28].

AFM observations, shown in Figure 1a, also confirm the hexagonal close-packed ordering with an average surface roughness of the film of approximately 1.4 nm.

**Figure 2:**
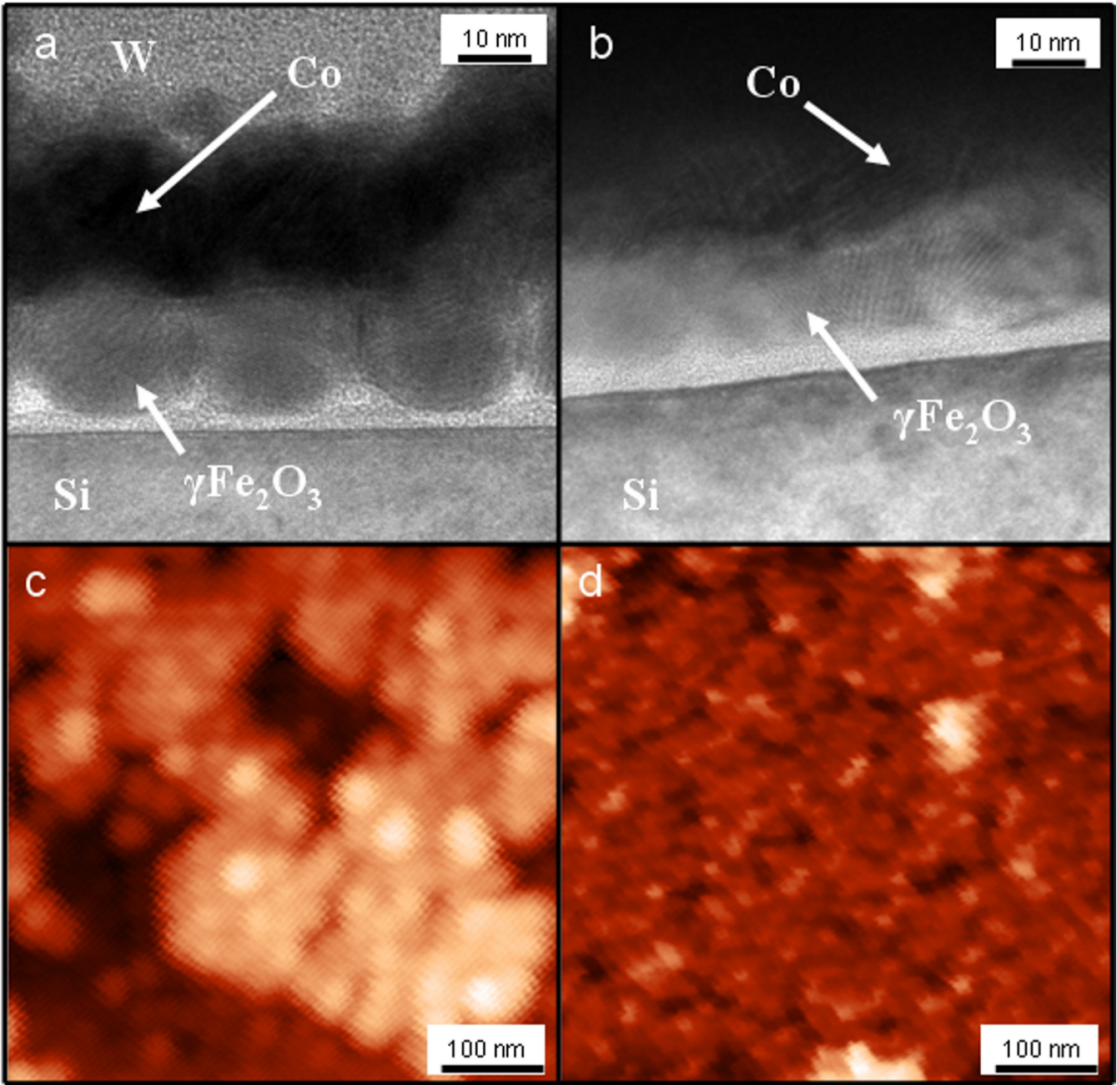
Top panel: High-resolution TEM cross-section images of non-ion-milled (a) and ion-milled (b) composite samples. Bottom panel: AFM images of the Co surface for the non-ion-milled (c) and ion-milled (d) samples.

Ion-milling carried out at the surface of the NPs removed the oleic acid layer, flattened the NPs at the top, and reduced the surface roughness prior to the deposition of a Co layer on top. Approximately, a 2 to 3 nm thick layer was removed from the surface during milling. Cross sectional TEM images of the samples are shown in Figure 2b. For comparison, a reference sample that has not undergone ion-milling is also depicted in Figure 2a. Without ion-milling, the Co layer replicates the topography of the NPs beneath, which is much less in the case of the Co layer on the ion-milled NPs.

AFM images of the two samples prepared with non-ion-milled (Figure 2c) and with ion-milled (Figure 2d) NPs are in good agreement with the TEM observation. In the former case the topography of the NPs is reflected on the Co surface, while, after milling the surface is flattened down with a reduction in the average roughness from 5.8 nm to 1.8 nm.

### Magnetic characterization

**Figure 3:**
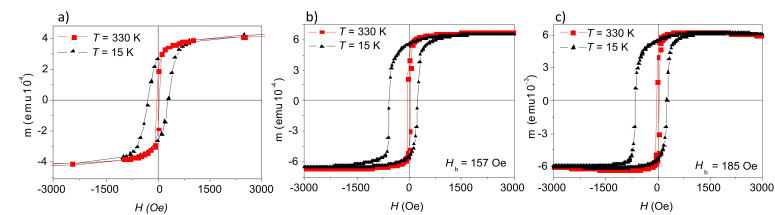
Magnetic hysteresis loops at 330 K and 15 K for a monolayer film of nanoparticles (a) and the composite nanoparticle/Co film non-ion-milled (b) and after ion-milling (c).

Magnetization hysteresis loops of a monolayer film, consisting of single phased maghemite NPs as detailed in the experimental section, are shown in Figure 3a. Hysteresis loops taken at 330 K and 15 K show the expected behavior of nanosized ferrimagnetic particles, i.e., symmetric loops, with a coercivity of *H_c_* = 280 Oe at 15 K and *H_c_*= 40 Oe at 330 K. The large increase in coercivity at low temperature is in agreement with previous reports and with the model of superparamagnetic (SPM) particles [29–30].

After the deposition of Co on top of the NP arrays, the *H_c_* at 15 K increases to 408 Oe and 455 Oe for the non-ion-milled (Figure 3b) and the ion-milled (Figure 3c) samples, respectively, while at 330 K the *H_c_* values with and without the Co layer are essentially the same. The interaction between the NPs and the Co layer becomes more pronounced at low temperatures and is expressed by a further increase of the coercivity and in a change of the shape of the hysteresis loop. In addition, it should be noted that, while the hysteresis loop for the NP monolayer is symmetric, the composite systems show a significant bias. It is important to note that the bias is only observed when the sample is field cooled, implying that its origin should be ascribed to an antiferromagnetic/ferromagnetic (AF/FM) coupling [31–35].

The magnetic exchange interaction between an AF and an FM layer can usually be observed as a horizontal shift of the magnetic hysteresis loop, when cooling the material from a temperature above the Néel temperature in an applied magnetic field. This offset is defined as exchange bias (EB) field, *H_b_* [32–33]. We find EB values at 15 K of *H_b_* = 157 Oe and 185 Oe for the non-ion-milled and the ion-milled system, respectively.

Since the system considered here is composed of single-phase ferrimagnetic maghemite NPs and a ferromagnetic Co thin film, it is necessary to account for the presence of an extra AF component. A possible explanation is that the Co layer is partially oxidized to AF CoO. The Co layer is capped with a protective Cu layer, and therefore, oxidation is more likely to occur at the particle/film interface by oxygen exchange from both the iron oxide and the organic oleic acid to the Co layer. In the event of oxygen exchange between the iron oxide nanoparticles and the Co layer, it is reasonable to expect a change in stoichiometry of the nanoparticles, at least at the surface level, close to the interface. However, it was not possible to verify this aspect, either from direct TEM images or to infer it from magnetic measurements. Further work is necessary to clarify this point. In any case, the EB is likely due to the exchange interaction between the AF CoO interfacial layer and the FM layer.

**Figure 4:**
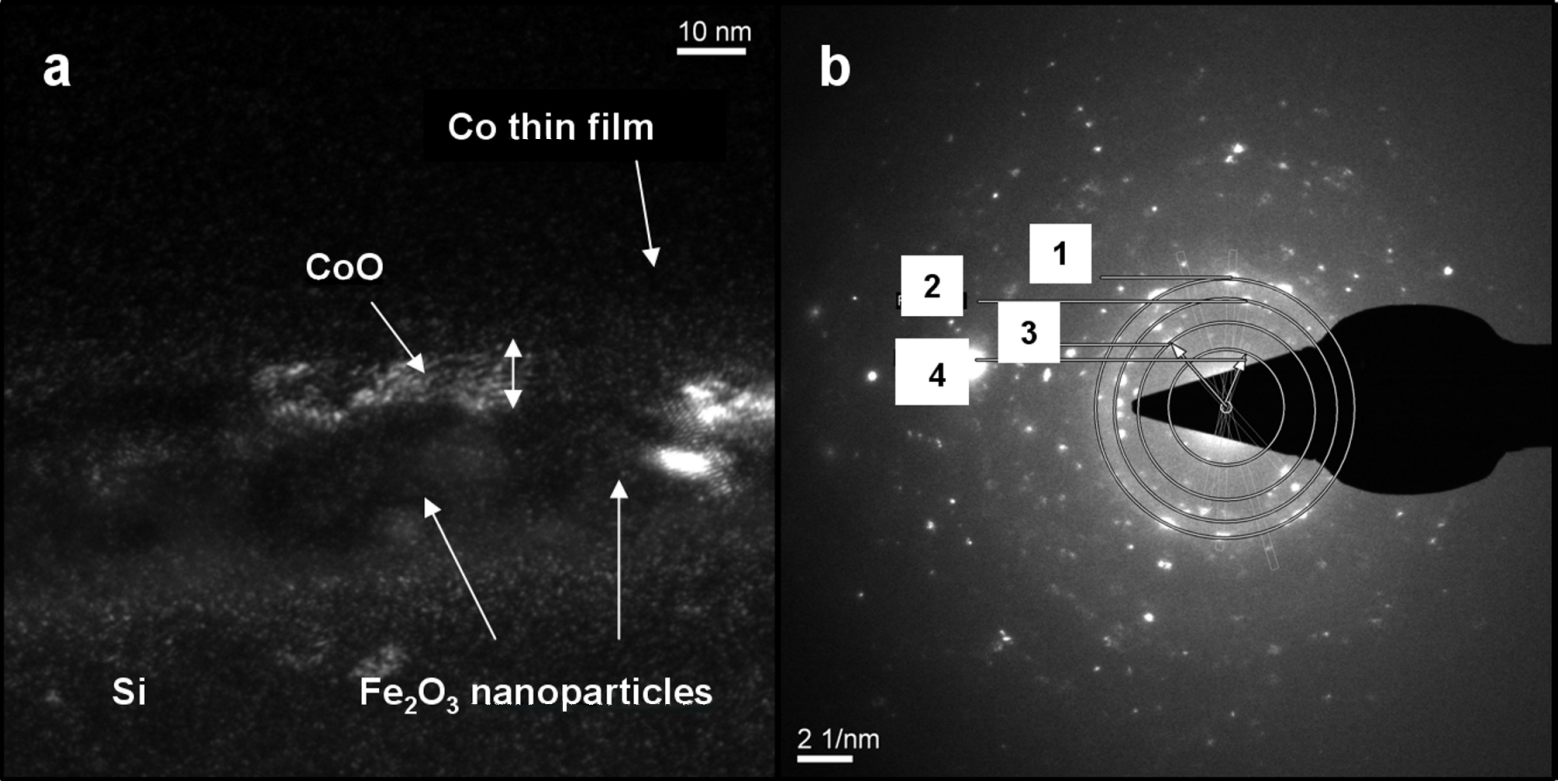
(a) Dark-field TEM image of the cross section NPs/thin-film system showing the CoO layer at the interface with NPs. (b) The corresponding diffraction pattern where the following phases are identified: 1) CoO (200), 2) Fe_2_O_3_ (311), 3) Si (111), 4) Fe_2_O_3_ (111).

This CoO layer is estimated to be between 1 to 4 nm thick. Although it was not possible to resolve such a CoO layer from the high-resolution TEM images (Figure 2), dark-field TEM images (Figure 4a) reveal the presence of a crystalline ~4 nm thick layer being well distinguishable from the Co layer, and thus attributed to the formation of an oxide phase in the Co film. The corresponding diffraction pattern shown in Figure 4b confirms the existence of a CoO crystal structure.

**Figure 5:**
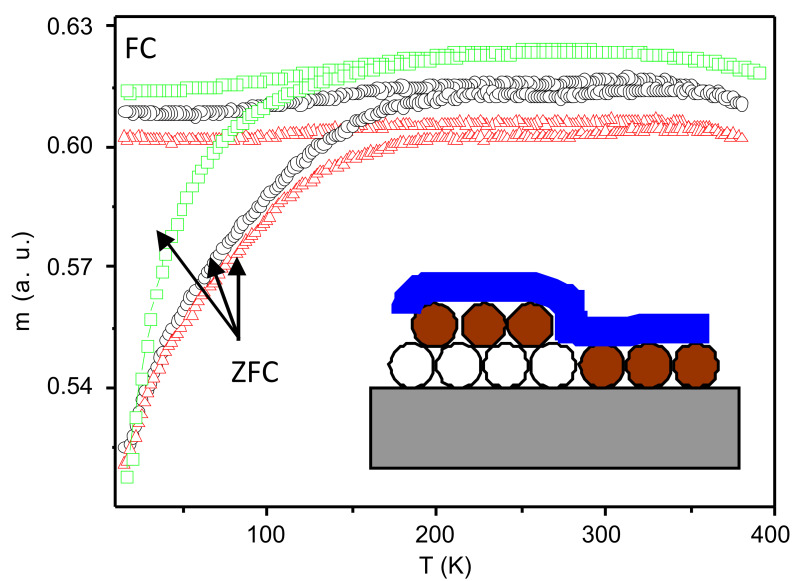
ZFC/FC magnetic moment vs temperature measured in 500 Oe for a NP monolayer (green squares), non-ion-milled composite (black circles) and ion-milled composite (red triangles), respectively. The curves are scaled for better clarity. The inset shows a schematic of the composite system. The Co film is depicted as a blue solid film and the NPs as circles, where two subsystems are marked: the open circles represent particles which are not in contact with the Co layer, and the filled brown circles are particles in contact with or near to the Co, respectively.

Further information about the magnetic behavior and in particular about the coupling effects between the NPs and the Co layer can be obtained from measurements of the magnetic moment vs temperature (Figure 5) after zero-field cooling (ZFC) and field cooling (FC).

Generally, the system is first cooled down from relatively high temperatures (here 380 K) in a zero field, then a magnetic field is applied and the ZFC curve is measured. The FC curve is usually obtained directly following the ZFC curve upon cooling in the same applied field.

Figure 5 shows m_ZFC_ and m_FC_ measured at 500 Oe for the three systems, i.e., the NP monolayer (green squares), non-ion-milled composite (black circles) and ion-milled composite (red triangles), respectively. The ZFC/FC curves for the NP monolayer show the regular behavior as expected from a SPM system, i.e., a peak in the ZFC curve marking the blocking temperature, *T*_b_ ≈ 250 K, of the system and the splitting of the ZFC and FC curves near *T*_b_. However, an important feature is the decrease of the magnetic moment in the FC curve for decreasing temperatures below *T*_b_. This trend has already been recognized as indicating a collective particle behavior, a so-called super-spin glass (SSG) state [21,24,27,36–37]. The peak temperature then marks the 'blocking temperature', however, not of individual NPs, but of the entire *interacting* monolayer of NPs.

When adding a Co layer on top of the NPs, the collective behavior of the NPs is partially inhibited as found from a more shallow dip in the FC curve. Interestingly, in the composite systems the ZFC/FC curves reveal the presence of two separate *T_b_* peaks, i.e., at ≈340 K and at ≈210 K and hence one above and one below, respectively, the blocking temperature of the NP monolayer.

There are two possible origins for the two peaks feature. In first place it might be due to the existence of two different NP subsystems as schematically depicted in the inset of Figure 5. The Co film does not cover all NPs equally, but only the top layer of NPs. In a 'monolayer' of NPs (that means one layer of particles on average) there exist not only holes and dislocations in the array but also areas with a second layer (see Figure 1). Hence, there will be NPs that are not in contact with the Co layer (open circles in the inset of Figure 5). Consequently, one might expect two magnetic subsystems, i.e., firstly NPs which are strongly magnetically coupled to the Co film or exchange biased to the CoO layer mentioned above at the NP-Co interface. These NPs are likely to produce an increased blocking temperature due to an increased energy barrier originating from the additional coupling.

Secondly, the other peak at lower temperatures is then due to NPs that are not in contact to the Co layer. These particles are weakly coupled to the other NPs by dipolar interactions. Because this subsystem consists of fewer particles than the entire NP ensemble, the collective blocking temperature of this smaller system will be reduced.

The second possible origin of the low-field peak might also arise from the CoO layer. It was in fact reported for a FM/AF coupled Fe_3_O_4_/CoO [38] and Fe/CoO [39] thin film systems, that the blocking temperature, in this case the temperature at which exchange bias between a FM and an AF thin film disappears, can occur at temperatures below the Néel temperature of CoO (~290 K), in the case of ultra thin films of CoO (less than 5 nm). Considering the thickness of the CoO film naturally grown in our system, it cannot be excluded that the peak at lower temperature in Figure 5 might be caused by the disappearance of the FM/AF coupling. In order to clarify this point further experimental work is necessary.

**Figure 6:**
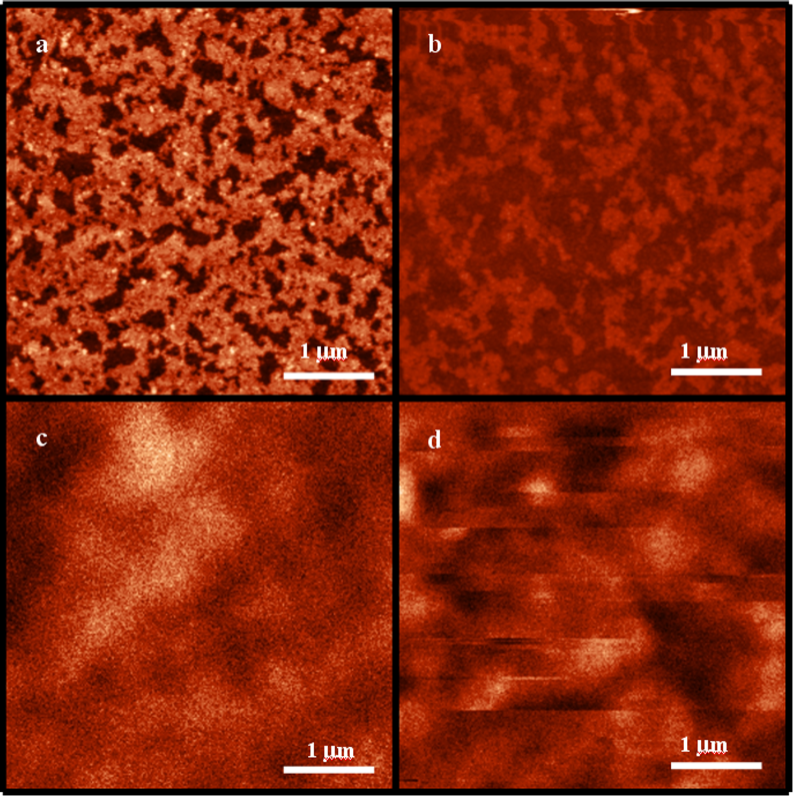
Top panel: AFM images of the Co surface for the non-ion-milled (a) and ion-milled (b) composite systems. Bottom panel: MFM images of the Co surface of non-ion-milled (c) and ion-milled (d) composite samples, taken with the sample in the remanent state after saturating at 1000 Oe.

MFM was used to investigate the surface domain structure of the composite materials with the sample in the remanent state (Figure 6c and Figure 6d). For comparison, the corresponding AFM images are also shown (Figure 6a and Figure 6b). In the case of the ion-milled system no well-defined magnetic domain structure at remanence can be recognized. The stray field of the MFM tip was found to modify the magnetic contrast during scanning (see Figure 6d). In order to rule out the possibility of artificial features from the tip or from surface impurities, different scans were performed after magnetic cycling. This effect was reproducible over multiple scans. Accounting for the better contact between particles and thin film and thus stronger magnetic coupling, this phenomenon can be due to the particles collecting the magnetic flux in a mechanism similar to that exerted by soft magnetic underlayers in perpendicular recording media [13]. This effect, however, becomes reduced with increasing interface roughness, as is the case for the non-ion-milled NPs. Here a sample-tip interaction was not observed (Figure 6c). A diffuse but stable domain structure in the Co layer is observed.

## Conclusion

Self assembled magnetic NP/thin film composites were prepared by a combination of spin-coating and ion-beam sputtering techniques. Ion-milling of the NP surface was used prior to Co sputtering for removing the oleic acid shell at the top of the NPs and to smooth out the interface prior to Co film deposition. This process was found to improve the coupling between the NPs and the Co layer. A shift of the hysteresis loop at low temperatures indicates an AF/FM exchange bias effect in the composite system, which is likely due to the formation of a CoO layer at the interface. The single NP layer exhibits a stretched blocking temperature, indicative of a collective behavior due to magnetic dipole interaction. The composite system shows two blocking temperatures: one above the temperature of the single NP layer, which likely is due to the interaction with the Co-layer, and one below, which we assign to small NP islands that are in contact with other NPs on top but not with the Co layer. Moreover, a 'soft magnetic underlayer' behavior of the ion-milled system was observed by MFM measurements. In conclusion, the contact of magnetic NPs with a closed thin magnetic film increases the blocking temperature of the system, i.e., it increases the potential well for thermal fluctuations. Composite systems also exhibit an enhanced coercivity and a change in the shape of the hysteresis loop at low temperature. The other two effects, exchange bias and a second lower blocking temperature, are extrinsic and depend on the particular system chosen.

## Experimental

Iron oxide NPs were prepared by thermal decomposition of metal-oleate complexes [40]. As-prepared, particles with mean diameter of 20 nm and 7% size distribution were coated with a ~2 nm thick layer of oleic acid and dissolved in toluene. The NP dispersion, with a concentration of approximately 50 mg of NPs per 5 ml of toluene, was spin-coated at 3000 rpm for 30 s on top of a Si(100) substrate with a natural oxide layer. As a result of the spin-coating process, approximately one monolayer of self-organized particles was formed having hexagonal closed-packed lateral order (see Figure 1). The samples were annealed at 170 °C for 20 min in air in order to obtain mainly single phase maghemite (γ-Fe_2_O_3_) NPs as reported in Ref. [41].

After heat treatment, the NP monolayer was ion-milled with neutralized Ar-ions for 4 min in order to flatten the NP array and remove the oleic acid layer. Finally, a thin cobalt film of 20 nm thickness was grown on top of the NPs by ion-beam sputtering from a Co target at 3.9 × 10^−4^ mbar with a base pressure of 1 × 10^−8^ mbar. To prevent oxidation of the Co surface, the sample was finally capped with a 3 nm thick layer of Cu. A reference composite sample was prepared for comparison, where the NPs were not ion-milled prior to the sputtering of Co.

The structure and topography of the samples were characterized by means of scanning electron microscopy (SEM) with a FEI Quanta FEG-SEM, transmission electron microscopy (TEM) with an analytical 200 kV FEG-TEM TECNAI F20 S-Twin instrument, atomic force and magnetic force microscopy (AFM, MFM) with an NT-MDT low temperature HV-Solver system. For cross sectional investigations of the composite film, TEM foils were extracted perpendicularly to the sample surface, by means of focused ion-beam technique, for which the sample had to be coated with an approximately 3 µm thick layer of tungsten. Magnetic measurements were performed by means of superconducting quantum interference device (SQUID) magnetometry (Quantum Design, MPMS) on sample areas of 7 × 7 mm^2^, in a temperature range between 15 and 380 K, with the field applied in the plane of the sample.
